# Factors Impacting the Telephone Follow‐Up Response Rate in Patients With Migraine Without Aura: A Cross‐Sectional Study of 1299 Females

**DOI:** 10.1155/prm/1347085

**Published:** 2026-09-21

**Authors:** Weinan Na, Junxia Zhao, Tingting Zhang, Yuanji Zhou, Hua Liu, Yinglu Liu, Zhao Dong, Shengyuan Yu, Xiaolin Wang

**Affiliations:** ^1^ Department of Neurology, The First Medical Center, Chinese PLA General Hospital, Beijing, China, 301hospital.com.cn; ^2^ School of Medicine, Nankai University, Tianjin, China, nankai.edu.cn

**Keywords:** female, migraine, response rate, telephone follow-up

## Abstract

**Background:**

The out‐of‐hospital management of migraineurs is an important part of migraine treatment. Telephone follow‐up is a commonly used method. To explore the factors impacting the response rate of follow‐up by telephone in migraineurs, 1299 female patients with migraine without aura (MwoA) from the headache outpatient database were followed.

**Methods:**

The corresponding information, including demographic factors, call time (morning, afternoon, or evening), call day (working day or weekend), and headache‐related factors was collected and analyzed.

**Results:**

Among the 1299 eligible female patients with MwoA, 821 (63.2%) responded (positive response, PR), and among them, 204 (24.8%) contacted us initiatively after follow‐up (feedback response, FR). Age < 45 (*p* = 0.034), calling in the morning (*p* = 0.005), and nonthrobbing headache (*p* = 0.037) were factors related to PR. Multivariate logistic analysis showed that age < 35 (OR 1.480, *p* = 0.007), 35 ≤ age < 45 (OR 1.466, *p* = 0.009), call in the morning (OR 1.487, *p* = 0.010), headache course ≥ 6 years (OR 1.363, *p* = 0.017), and no aggravation by activity (OR 1.525, *p* = 0.048) were related to PR. The analysis of factors influencing FR indicated that age < 40 (*p* < 0.001), high education level (*p* = 0.043), age at onset < 25 (*p* < 0.001), and more accompanying symptoms (*p* = 0.050) were related to FR. The multivariate logistic analysis suggested that living in a first‐tier city (OR 1.977, *p* = 0.015), age at onset < 25 (OR 1.499, *p* = 0.025), age < 40 (OR 1.531, *p* = 0.025), and accompanying symptom score = 3 (OR 1.787, *p* = 0.039) were related to FR.

**Conclusions:**

Age, headache course, call time, and headache‐related factors were associated with the response rate for migraineurs. Residence in first‐tier cities was associated with a higher observed probability of feedback response. Age, follow‐up time, city of residence, and advance notification may be factors worth considering when designing a personalized follow‐up plan to optimize the response rate among female migraineurs.

## 1. Introduction

Migraine is a frequent neurological disorder with estimates of globalprevalence reaching 14%–15% [1]. It is the primary headache with the highest disability in clinical practice [2, 3]. Although migraine is a disease that is difficult to cure [4, 5], it can be prevented and controlled [6, 7]. Disease education for migraineurs is an important method against migraine chronification and medicine overuse. To help migraineurs build a comprehensive understanding of their migrainous disorder and enhance their confidence in fighting it, regular follow‐up is necessary. Telephone‐based interviews have shown good reliability in headache diagnosis [8] and have been used for migraine follow‐up to track clinical changes [9]. However, the factors affecting the success of telephone follow‐up in migraineurs have never been systematically evaluated. This is a secondary analysis of a main study focusing on the clinical feature research in female migraineurs without aura (MwoA) [10]. We aim to figure out three questions: (a) What are the differences in characteristics of responders vs. non‐responders, including demographic factors and headache features; (b) Among the patients consisting mainly of women of reproductive age, does the calling time (weekdays vs. weekend, daytime vs. evening) influence the telephone follow up response; (c) What are the characteristics of patients who contacted the researcher initiatively after the follow‐up and expressed strong willingness to participate in future follow‐up and headache clinic research. These analyses will provide a real‐world research result helping improve the follow‐up response evaluating method.

## 2. Methods

### 2.1. Patients

This is a secondary analysis of a main study on female MwoA patients [10]. As a secondary analysis of the main study, although our analytical strategy was determined prior to data inspection, we did not prespecify directional hypotheses for this exploratory analysis. All the patients’ personal and headache‐related information was recorded at the first visit to the headache center. Female patients with MwoA from January 2015 to January 2020 were initially selected in the study and were screened and reassessed by at least two qualified and experienced headache experts to exclude atypical migraineurs. The MwoA criteria were from the International Classification of Headache Disorders, the third edition (ICHD‐III) [2], which was published by the International Headache Society in 2018. Patients who could not be contacted for objective reasons, including wrong recorded number, number cancellation, and phone shutdown (contacted at least three times), were excluded from the study. Patients who agreed to participate in the follow‐up were classified as positive response (PR) patients. Patients who refused to participate in the follow‐up, rejected the phone, or did not answer the phone three times were classified as negative response (NR) patients. Patients who contacted us initiatively after follow‐up were classified as feedback response (FR) patients.

### 2.2. Follow‐Up Procedures and Data Collection

Variables included age, headache course, city of residence (first‐tier cities and non‐first‐tier cities, according to the National Bureau of Statistics [11], specifically, first‐tier cities refer to Beijing, Shanghai, Guangzhou, and Shenzhen [12, 13]), education level (four degrees: below junior high school, junior high school, senior high school, and above high school), call day (working days and weekend), call time (morning, afternoon and evening), headache days per month, Number Rating Scales for pain (NRS score), throbbing headache, aggravation by activity, accompanying symptoms (nausea, vomiting, photophobia, phonophobia), and family history. To evaluate the degree of accompanying symptoms, the four symptoms listed below were assigned weights, and the cumulative sum was taken as the accompanying symptom score, which was calculated as follows: accompanied by nausea (0, no; 1, yes), vomiting (0, no; 1, yes), photophobia or phonophobia (0, no; 1, yes). To evaluate the characteristics of the pain, based on the migraine criteria, headache‐related factors were assigned weights as follows: unilateral headache (0, no; 1, yes), throbbing headache (0, no; 1, yes), NRS [0, mild pain (NRS 1–3); 1, moderate to severe pain (NRS 4–10)], aggravation by activity (0, no; 1, yes), and the cumulative sum was taken as the pain feature score.

Our follow‐up group included two experienced headache experts, three nurses, and two graduate students who specialized in headaches. A standardized follow‐up operating procedure included a total of three phone call attempts in this study. The first phone call was a routine contact. After that, those who were out of contact because of rejected calls, unanswered calls, and shutdowns would undergo the second phone call procedure. Those who were still out of contact would undergo the third call procedure but would be informed 2 h in advance by sending a short message explaining the follow‐up intention.

### 2.3. Data Availability and Ethical Approval

All participants were informed of their right and the purpose of the follow‐up by telephone. Oral informed consent was obtained from all subjects due to the data collection nature of the study. The study protocol was approved by the Ethics Committee of Chinese PLA General Hospital (2020263) and complied with China’s regulations and Guidelines for Good Clinical Practice. We will share anonymized data upon reasonable request from any qualified investigator. This study meets the STROBE guidelines for cross‐sectional human observational studies.

### 2.4. Statistical Analysis

SPSS23.0 software was used for statistical analysis. The Mann–Whitney *U* test was performed for non‐normally distributed continuous data and ranked data. Chi‐square tests or Fisher exact tests were performed for categorical data. Variables with *p* < 0.1 in univariate analysis and other variables of interest [14–16] were included in the binary logistic regression (enter method). The assumptions of binary logistic regression were assessed prior to the final analysis. Specifically, we examined multicollinearity (variance inflation factor, VIF < 10), verified adequate events‐per‐variable ratios (> 10), and evaluated model calibration using the Hosmer–Lemeshow goodness‐of‐fit test. Multiple imputation was used to impute education level with missing values before multivariate analysis. We conditioned the estimation of education level on demographic factors and headache‐related factors (age, city of residence, headache course, headache days per month, NRS score for pain, throbbing headache, aggravation by activity, accompanying symptoms, and family history). We evaluated the interaction effect between call day, call time, and city of residence by adding it to the regression model. In the above statistical analysis, *p* < 0.05 was considered to be statistically significant.

## 3. Results

**FIGURE 1:**
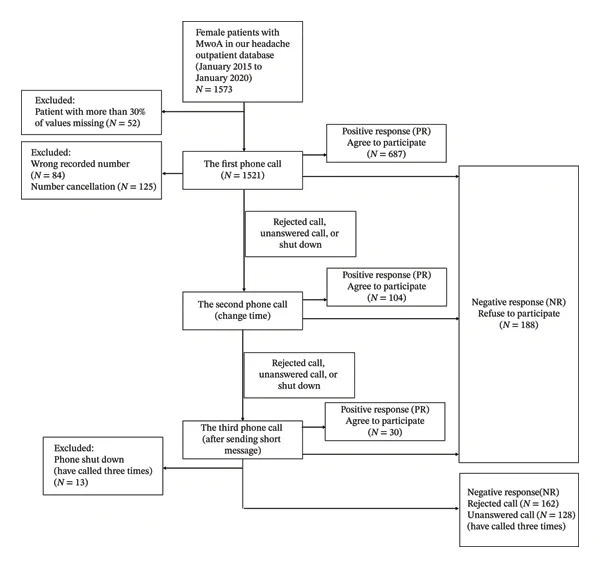
Flowchart of the study. A total of 1299 female patients with migraine without aura were included in the study. The chart shows the screening details and the number of patients in the three rounds of phone calls (1299 = 1573−52−84−125−13).

There were 1573 female patients who fulfilled the diagnostic criteria of MwoA. Among them, 274 were excluded from the study for large missing values (> 30% missing values in medical record variables, *n* = 52) and lost connection for objective reasons (*n* = 222), including wrong recorded number (*n* = 84), number cancellation (*n* = 125), and consistent telephone shutdown three times (*n* = 13). Therefore, a total of 1299 female MwoA patients were enrolled in the study (Figure 1). Among these 1299 eligible female patients, 821 (63.2%) were PR patients. A total of 687 (52.9%) patients were successfully followed through the first phone call. A total of 104 (8.0%) patients were contacted by changing the follow‐up time at the second call procedure. For the last call procedure, by sending a message in advance, 30 (2.3%) patients responded. For the 478 (36.8%) NR patients, 188 patients (39.3%) refused to participate, 162 patients (33.9%) rejected the phone, and 128 patients (26.8%) did not answer the phone. Furthermore, among the 821 PR patients, 204 patients (24.8%) contacted us initiatively after the follow‐up through the researcher’s social media account, which were FR patients.

Univariate analysis was performed to analyze the factors impacting the follow‐up response (Table 1). Age < 45 (*p* = 0.034), calling in the morning (*p* = 0.005), and nonthrobbing headache (*p* = 0.037) were related to a PR. For the logistic binary regression model, the VIF ranged from 1.004 to 3.046, the events‐per‐variable ratio was 102.625, and the Hosmer–Lemeshow test showed adequate fit (*χ* ^2^ = 5.594, *p* = 0.693). Binary logistic regression analysis showed that younger age (< 35 and 35‐45), call in the morning, long headache course (≥ 6 years), nonthrobbing headache, and no aggravation by activity were related to PR (Table 2).

**TABLE 1:** Univariate analysis between PR and NR.

Variables	*N* (%)		*p*
	NR (*n* = 478)	PR (*n* = 821)	
Age (year)			0.034^∗^
< 35	154 (32.2%)	287 (35.0%)	
35–45	130 (27.2%)	259 (31.5%)	
> 45	194 (40.6%)	275 (33.5%)	
City of residence			0.151
Non‐first‐tier city	325 (68.0%)	526 (64.1%)	
First‐tier city	153 (32.0%)	295 (35.9%)	
Education level			0.267
Junior high school and below	111 (35.7%)	170 (32.0%)	
Senior high school and above	200 (64.3%)	362 (68.0%)	
Call day			0.410
Working day	263 (55.0%)	471 (57.4%)	
Weekend	215 (45.0%)	350 (42.6%)	
Call time			0.005^∗∗^
Morning	200 (41.8%)	410 (49.9%)	
Afternoon or evening	278 (58.2%)	411 (50.1%)	
Age at onset			0.576
< 25	179 (37.4%)	347 (42.3%)	
≥ 25	299 (62.6%)	474 (57.7%)	
Headache course (years)			0.079
< 6	159 (33.3%)	235 (28.6%)	
≥ 6	319 (66.7%)	586 (71.4%)	
Headache days per month			0.426
< 1	42 (8.8%)	56 (6.8%)	
1–15	317 (66.3%)	560 (68.2%)	
> 15	119 (24.9%)	205 (25%)	
Unilateral headache			0.617
No	245 (51.3%)	409 (49.8%)	
Yes	233 (48.7%)	412 (50.2%)	
NRS for pain			0.301
0–6	151 (31.6%)	237 (28.9%)	
7–10	327 (68.4%)	584 (71.1%)	
Throbbing headache			0.037^∗^
No	217 (45.4%)	422 (51.4%)	
Yes	261 (54.6%)	399 (48.6%)	
Aggravation by activity			0.087
No	67 (14.0%)	133 (16.2%)	
Less than half	82 (17.2%)	106 (12.9%)	
More than half	329 (68.8%)	582 (70.9%)	
Pain feature score^b^			0.166^c^
2	163 (34.1%)	304 (37.0%)	
3	211 (44.1%)	363 (44.2%)	
4	104 (21.8%)	154 (18.8%)	
Accompanied by nausea			0.299
No	49 (10.3%)	70 (8.5%)	
Yes	429 (89.7%)	751 (91.5%)	
Accompanied by vomiting			0.214
No	174 (36.4%)	271 (33.0%)	
Yes	304 (63.6%)	550 (67.0%)	
Accompanied by photophobia			0.545
No	168 (35.1%)	275 (33.5%)	
Yes	310 (64.9%)	546 (66.5%)	
Accompanied by phonophobia			0.325
No	124 (25.9%)	193 (23.5%)	
Yes	354 (74.1%)	628 (76.5%)	
Accompanying symptom score^a^			0.097^c^
1	89 (18.6%)	120 (14.6%)	
2	151 (31.6%)	262 (31.9%)	
3	238 (49.8%)	439 (53.3%)	
Family history			0.210
No	260 (54.4%)	417 (50.8%)	
Yes	218 (45.6%)	404 (49.2%)	
Chronic headache^d^			
No	363 (75.9%)	621 (75.6%)	0.903
Yes	115 (24.1%)	200 (24.4%)	
Medication overuse^e^			
No	398 (83.3%)	687 (83.7%)	0.846
Yes	80 (16.7%)	134 (16.3%)	

*Note:* PR (positive response) patient refers to those who agreed to participant in the follow‐up; NR (negative response) patient refers to those who refused to participate and who rejected the phone nor did not answer the phone for three times.

^∗^ *p* < 0.05.

^∗∗^ *p* < 0.01.

^a^Accompanying symptom score is the cumulative sum of the following variable: accompanied by nausea = 1, accompanied by vomiting = 1, accompanied by photophobia or phonophobia = 1.

^b^Pain feature score is the cumulative sum of the following variables: unilateral headache = 1, NRS (4–10) = 1, throbbing headache = 1, aggravation by activity = 1.

^c^Mann–Whitney *U* test (Chi‐square test if not marked).

^d^Chronic headache: headache occurring on 15 or more days per month for more than 3 months (at least 8 days per month has the features of migraine headache).

^e^Medication overuse: headache occurring on ≥ 15 days per month and regular overuse for more than 3 months of one or more drugs that can be taken for acute and/or symptomatic treatment.

**TABLE 2:** Binary logistic regression of influencing factors for PR.

Variables	OR value (95% CI)	*p*
Age (years)		
≥ 45	—	—
35–45	1.466 (1.101, 1.954)	0.009^∗∗^
< 35	1.480 (1.115, 1.964)	0.007^∗∗^
Call time		
Afternoon or evening	—	—
Morning	1.487 (1.093, 2.022)	0.010^∗^
Call day		
Working day	—	—
Weekend	0.847 (0.598, 1.198)	0.348
Call time × call day	1.144 (0.719, 1.821)	0.571
Headache course		
< 6	—	—
≥ 6	1.363 (1.057, 1.757)	0.017^∗^
Throbbing headache		
Yes	—	—
No	1.361 (1.078, 1.718)	0.010^∗^
Aggravation by activity		
Less than half	—	—
More than half	1.346 (0.973, 1.862)	0.073
No	1.525 (1.004, 2.315)	0.048^∗^
Accompanying symptom score^a^		
1	—	—
2	1.271 (0.900, 1.796)	0.173
3	1.293 (0.935, 1.788)	0.121

*Note:* PR (positive response) patient refers to those who agreed to participant in the follow‐up. × interaction effect.

^∗^ *p* < 0.05.

^∗∗^ *p* < 0.01.

^a^Accompanying symptom score is the cumulative sum of the following variable: accompanied by nausea = 1, accompanied by vomiting = 1, accompanied by photophobia or phonophobia = 1.

Among the 821 PR patients, 204 (24.8%) patients contacted us initiatively (FR) after the follow‐up. Univariate analysis of factors influencing FR showed that age < 40 (*p* < 0.001), high education level (*p* = 0.043), age at onset < 25 (*p* < 0.001), and high accompanying symptoms score (*p* = 0.050) were related to FR (Table 3). For the logistic binary regression model, the VIF ranged from 1.047 to 2.907, the events‐per‐variable ratio was 18.54, and the Hosmer–Lemeshow test showed adequate fit (*χ* ^2^ = 7.309, *p* = 0.504). Binary logistic regression analysis showed that living in a first‐tier city (OR 1.977, *p* = 0.015), age at onset < 25 (OR 1.499, *p* = 0.025), patient age < 40 (OR 1.531, *p* = 0.025), and accompanying symptom score = 3 (OR 1.787, *p* = 0.039) were related to FR (Table 4).

**TABLE 3:** Univariate analysis on FR.

Variables	*N* (%)		*p*
	Non‐FR (*n* = 617)	FR (*n* = 204)	
Age (years)			< 0.001^∗∗^
< 40	286 (46.4%)	127 (62.3%)	
≥ 40	331 (56.3%)	77 (37.7%)	
City of residence			0.072
Non‐first‐tier city	406 (65.8%)	120 (58.8%)	
First‐tier city	211 (34.2%)	84 (41.2%)	
Education level			0.043^∗^
Junior high school or below	139 (34.2%)	31 (24.6%)	
Senior high school or above	267 (65.8%)	95 (75.4%)	
Call day			0.072
Working day	365 (59.2%)	106 (52.0%)	
Weekend	252 (40.8%)	98 (48.0%)	
Call time			0.531
Morning	312 (50.6%)	98 (48.0%)	
Afternoon or evening	305 (49.4%)	106 (52.0%)	
Age at onset (years)			< 0.001^∗∗^
< 25	238 (38.6%)	109 (53.4%)	
≥ 25	379 (61.4%)	95 (46.6%)	
Headache course (years)			0.335
< 6	182 (29.5%)	53 (26.0%)	
≥ 6	435 (70.5%)	151 (74.0%)	
Headache days per month			0.800
< 1	43 (7.0%)	13 (6.4%)	
1–15	417 (67.6%)	143 (70.1%)	
> 15	157 (25.4%)	48 (23.5%)	
Unilateral headache			0.385
No	302 (48.9%)	107 (52.5%)	
Yes	315 (51.1%)	97 (47.5%)	
NRS for pain			0.078
0–6	188 (30.5%)	49 (24.0%)	
7–10	429 (69.5%)	155 (76.0%)	
Throbbing headache			0.764
No	319 (51.7%)	103 (50.5%)	
Yes	298 (48.3%)	101 (49.5%)	
Aggravation by activity			0.996
No	100 (16.2%)	33 (16.2%)	
Less than half	80 (13%)	26 (12.7%)	
More than half	437 (70.8%)	145 (71.1%)	
Pain feature score^b^			0.326^c^
2	222 (36.0%)	82 (40.2%)	
3	282 (45.7%)	81 (39.7%)	
4	113 (18.3%)	41 (20.1%)	
Accompanied by nausea			0.909
No	53 (8.6%)	17 (8.3%)	
Yes	564 (91.4%)	187 (91.7%)	
Accompanied by vomiting			0.152
No	212 (34.4%)	59 (28.9%)	
Yes	405 (65.6%)	145 (71.1%)	
Accompanied by photophobia			0.077
No	217 (35.2%)	58 (28.4%)	
Yes	400 (64.8%)	146 (71.6%)	
Accompanied by phonophobia			0.058
No	155 (25.1%)	38 (18.6%)	
Yes	462 (74.9%)	166 (81.4%)	
Accompanying symptom score^a^			0.050^c^
1	101 (16.4%)	19 (9.3%)	
2	195 (31.6%)	67 (32.8%)	
3	321 (52.0%)	118 (57.8%)	
Family history			0.364
No	319 (51.7%)	98 (48.0%)	
Yes	298 (48.3%)	106 (52.0%)	
Chronic headache^d^			
No	465 (75.4%)	156 (76.5%)	0.750
Yes	152 (24.6%)	48 (23.5%)	
Medication overuse^e^			
No	511 (82.8%)	176 (86.3%)	0.247
Yes	106 (17.2%)	28 (13.7%)	

*Note:* FR (feedback response) patient refers to those who contacted us initiatively after follow‐up.

^∗^ *p* < 0.05.

^∗∗^ *p* < 0.01.

^a^Accompanying symptom score is the cumulative sum of the following variable: accompanied by nausea = 1, accompanied by vomiting = 1, accompanied by photophobia or phonophobia = 1.

^b^Pain feature score is the cumulative sum of the following variables: unilateral headache = 1, NRS (4–10) = 1, throbbing headache = 1, aggravation by activity = 1.

^c^Mann–Whitney *U* test (Chi‐square test if not marked).

^d^Chronic headache: headache occurring on 15 or more days per month for more than 3 months (at least 8 days per month has the features of migraine headache).

^e^Medication overuse: headache occurring on ≥ 15 days per month and regular overuse for more than 3 months of one or more drugs that can be taken for acute and/or symptomatic treatment.

**TABLE 4:** Binary logistic regression of influencing factors for FR.

Variables	OR value (95% CI)	*p*
City of residence		
Non‐first‐tier city	—	
First‐tier city	1.977 (1.140, 3.426)	0.015^∗^
Call time		
Morning	—	—
Afternoon or evening	0.939 (0.556, 1.586)	0.814
NRS for pain		
0–6	—	—
7–10	1.421 (0.974, 2.074)	0.069
Age at onset (years)		
≥ 25	—	—
< 25	1.499 (1.053, 2.134)	0.025^∗^
Education level		
Junior high school or below	—	—
Senior high school or above	0.970 (0.638, 1.475)	0.888
Age (years)		
≥ 40	—	—
< 40	1.531 (1.056, 2.2201)	0.025^∗^
Accompanying symptom score^a^		
1	—	—
2	1.698 (0.952, 3.029)	0.073
3	1.787 (1.030, 3.100)	0.039^∗^
Call day		
Working day	—	—
Weekend	1.066 (0.616, 1.844)	0.820
Call day × call time	1.752 (0.906, 3.386)	0.096
City of residence × call day	0.732 (0.372, 1.438)	0.365
City of residence × call time	0.620 (0.317, 1.212)	0.162

*Note:* FR (feedback response) patient refers to those who contacted us initiatively after follow‐up. × interaction effect.

^∗^ *p* < 0.05.

^a^Accompanying symptom score is the cumulative sum of the following variable: accompanied by nausea = 1, accompanied by vomiting = 1, accompanied by photophobia or phonophobia = 1.

## 4. Discussion

Telephone interviews are widely used in migraine research to collect clinical outcome measures [17–21], capture patient‐reported subjective experiences and comorbidity data [22–25], and support epidemiological surveys [26]. They are particularly advantageous when in‐person visits are impractical—for example, during pandemic restrictions [9], when studying special populations such as pregnant women [20], or in large multinational multicenter studies that require consistent data collection across diverse sites [26]. Beyond using telephone interviews to collect data, some studies have also evaluated the telephone interview itself as an instrument in migraine patients. As a diagnostic tool, semistructured telephone interviews appear to be more reliable than self‐report questionnaires for accurately diagnosing headache disorders [8]. As a follow‐up and intervention tool, telephone interview improves medication adherence [27], yields higher satisfaction than business‐as‐usual (BAU) treatment [28], and is safe and effective for migraine patients compared with outpatient visits [29].

Contemporary digital strategies, including text messaging and online platforms, are increasingly used alongside traditional telephone follow‐up. The comparison between online and telephone modes varies by context. In population surveys, online approaches (web‐based) enable faster recruitment and lower costs per interview [30]. In patient follow‐up, however, findings differ: telephone achieved higher response rate in trauma study with 6‐month follow‐up [31], whereas online (via QR code) achieved higher attendance 1‐day postoperative follow‐up in an ophthalmic surgery study [32]. This suggests that the optimal mode depends on follow‐up duration and patient population. Telephone follow‐up achieved similar response rates to text messaging [33], but was associated with substantially lower rates of postoperative minor symptom reporting and reconsultations [34].

Given the importance of follow‐up in the management of migraine, this study is appropriate, and would probably provide clues to improve the follow‐up strategy. In this study, 222 patients were initially excluded for objective reasons, including wrong number recorded, number being canceled, and phone shutdown (call at least three times). This result suggests that doctors who receive the first visit of migraineurs should emphasize accessibility through the telephone number filled and recommend leaving at least two valid telephone numbers written in the data form.

In the second round of phone calls, by changing the call time, the number of PR patients increased by 104 (8.0%). By sending a short message in advance at the third round, 30 (2.3%) cases responded positively. After three rounds of phone calls, 821 (63.2%) patients were successfully followed up. Although we did not design two separate cohorts to directly compare the effects of different call times or the use of advance text messages on response rates, the results here provide some implications for how to increase the successful follow‐up rate by phone. Previous studies have reported multiple methods to improve response rates in both general population and patient follow‐up studies. A Cochrane systematic review [35], which summarized many methods to increase response to postal and electronic questionnaires, found that monetary incentives, pre‐notification, shorter questionnaires, placing clinical outcome questions last, including a picture in an email, and using a more interesting topic are among the most effective methods for increasing response rates. Regarding the effect of call timing on response rate, a previous study [36] among the guardians of newborns who failed deafness gene screening found no significant difference in adherence rates between weekend and weekday calls, which was consistent with this study. A possible reason may be that electronic communication has blurred the work–weekend boundary. That study [36] also showed higher adherence for afternoon calls on weekdays and for morning calls on weekends. This partly aligns with our finding that morning calls were associated with PRs on both weekdays and weekends (no interaction between the call day and call time). This suggests that on weekdays, work‐time calls are more likely answered for young parents (their study) and middle‐aged females (ours), likely because evenings are occupied with childcare. Regarding the effect of advanced text message on response rate, previous studies have shown that reminders in various forms (e.g., short message [37, 38], email [38], and letter [39]) can improve response rates. Our preliminary finding aligns with this evidence, suggesting that advanced text message may improve participation. This effect may stem partly from participants’ reluctance to answer unfamiliar calls due to privacy concerns, and partly from the reminder’s direct prompting function that draws attention to the task.

By analyzing the influencing factors for response rate, we found that age, call time, headache course, throbbing headache, and aggravation by activity were associated with the response rate of female MwoA patients.

In our study, compared with middle‐aged patients, young patients were more likely to positively respond (age < 35 vs. age ≥ 45, OR 1.480, *p* = 0.007; age 35–45 vs. age ≥ 45, OR 1.466, *p* = 0.009). A study of patients with headache regarding their willingness to use teleconsultation showed that patients aged 25–54 years were significantly more accepting of teleconsultation [40], which is similar to our findings. The reason is probably that younger people are more familiar with the technology and more willing to use devices to report their health data. Our result seems opposite to previous studies among nontumor postsurgery patients [41, 42], where young age was related to lost‐to‐follow‐up, suggesting that the impact of age on follow‐up response may vary across different disease types.

In this study, long‐course patients (≥ 6 years) were more likely to achieve PR than short‐course patients (< 6 years) (OR 1.363, *p* = 0.017). Headache‐related disability is a well‐documented burden among individuals with migraine [43, 44]. The longer the headache course, the greater the cumulative damage to daily life, which may in turn strengthen patients’ motivation to seek consistent medical management. This reasoning is in line with previous findings that longer headache duration is associated with better adherence to medication treatment [45]. Nonthrobbing headache had a relationship with PR (OR 1.361, *p* = 0.010), which was unexpected. Although statistically significant, this finding lacks a clear clinical rationale and should be regarded as exploratory. While our main research [10] showed that nonthrobbing headache had a relationship with motion disorder, sleep disorder, medicine overuse, and frequent headache—associations that may provide some rationale for our current finding—the observed relationship between nonthrobbing headache and PR requires further investigation. In our study, the absence of activity‐related aggravation was associated with better telephone follow‐up response—a finding that seems counterintuitive. One possible interpretation is that the absence of activity‐related aggravation may reflect fewer identifiable pain cues, which might prompt them to seek guidance via telephone follow‐up, though this remains a hypothesis requiring validation.

Among the 821 PR patients, 204 patients (24.8%) contacted the researchers initiatively after the follow‐up by adding the researcher’s social media account. We believe that this behavior itself is an indication of a high willingness to follow up. By analyzing related factors, we found that city of residence, the age of migraine onset, and age of the patient were associated with this intention. First‐tier cities tend to have more developed economies, more abundant medical resources, and more advanced medical technology. Residents of these cities also appeared more accepting of clinical research and telemedical consultation. This suggests that patients from more developed residences tend to have higher adherence to follow‐up regimens. This pattern is consistent with previous studies reporting urban–rural differences in medication adherence [46, 47] and income‐related disparities in migraine care, including the prescription of novel agents [48]. However, given the cross‐sectional design, the observed associations may be influenced by unmeasured confounders such as healthcare accessibility, digital literacy, socioeconomic status, and prior healthcare experiences. Additionally, young migraineurs are more accustomed to smartphones, and therefore may easily provide health data via such devices.

This study is a secondary analysis of a parent study conducted at a single tertiary headache center in China [10]. The original cohort comprised female MWoA patients who underwent rigorous diagnostic verification by headache specialists, which supports the quality of our data. Nevertheless, the single‐center design, the female‐only sample, and the restriction to variables collected in the parent study should be considered when interpreting our results.

### 4.1. Limitations

This study has several shortcomings. First, the relatively long study period means that patients’ headache‐related characteristics may have changed by follow‐up, and election bias is also a concern because 274 patients were excluded and may differ systematically from the analyzed cohort. Thus, our findings regarding response rates and their associated factors may have limited generalizability to the broader migraine population. Second, our results indicated that some negative responders may become positive responders after receiving the text message. A randomized controlled trial is necessary to provide high‐quality evidence for the contribution of sending a message in advance in telephone follow‐up. Third, as this study was an additional study from a main study [10] specified to female MwoA patients, male patients were excluded, which may add to the selection bias. Although the female‐predominant nature of migraine (female‐to‐male prevalence ratio = 2:1 in China [49]) supports the clinical relevance of studying women, our findings from a single‐sex, single‐subtype cohort (women with MwoA) should not be generalized to men or to patients with other migraine phenotypes without validation, as telephone follow‐up success is influenced by diverse demographic, social, and disease‐related factors. Additionally, our interpretation of the FR as a marker of willingness to participate in future follow‐up should be viewed with caution, as this behavior may also be influenced by factors such as digital literacy, personality, and socioeconomic status. Future studies may consider using more comprehensive questionnaire to assess willingness to participate, alongside the barriers and facilitators influencing such decisions, as previously applied in other settings such as cardiac telerehabilitation [50] and ophthalmic clinical trials [51]. The aim of this paper is not to explore the impact of telephone follow‐up on patients’ treatment, outcomes, and satisfaction, and the topic has been investigated in a Danish tertiary headache center [28]. Furthermore, examining those issues requires more than one follow‐up visit to dynamically observe changes in patient symptoms and medication usage, which can be further discussed in our subsequent studies. Another limitation is that we recorded the call attempt number for successful follow‐ups, but for those who refused to participate, we only had the total count, without knowing the specific call attempt for each refusal. Consequently, we were unable to examine whether the number of call attempts influenced the likelihood of refusal, or whether refusal patterns differed across call attempts. Given that our specific hypotheses were exploratory and not prespecified, and that we did not apply stringent corrections for multiple comparisons, our findings should be interpreted as hypothesis‐generating rather than confirmatory.

## 5. Conclusions

Telephone is a fast and efficient method for follow‐up. However, the response rate is affected by many factors. To improve the response rate, stating the purpose and significance when contact information is obtained, personalizing the follow‐up time according to the age of the patients, and sending a precall message informing the purpose of the call could be considered as potentially useful strategies. For female MwoA patients, less than 45 years old and with a headache course longer than 6 years were related to a high response rate. Young patients and patients living in first‐tier cities were more likely to initiate subsequent contact. These results suggest that a personalized strategy may be beneficial to migraineur follow‐up.

**Nomenclature**
MwoA: Migraine without aura
ICHD‐III: The International Classification of Headache Disorders, the third edition
NRS: Number rating scale
PR: Positive response
NR: Negative response
FR: Feedback response

## Author Contributions

Weinan Na and Junxia Zhao analyzed the data, drafted the manuscript, and interpreted the results.

Junxia Zhao, Tingting Zhang, and Yuanji Zhou performed the follow‐up and organized the data.

Zhao Dong, Shengyuan Yu, Hua Liu, and Yinglu Liu provided insightful comments on the study design and analysis.

Xiaolin Wang designed the work, revised the manuscript, interpreted the results, and accountable for all aspects of work.

## Funding

This study was funded by National Key Research and Development Program of China (2023YFC2508706 to Xiaolin Wang), Logistics Support Research Project of China (grant 145BHQ090003000X12 to Shengyuan Yu), and the National Natural Science (grant 82271242 to Shengyuan Yu, 82101288 to Yinglu Liu).

## Disclosure

All authors read and approved the final manuscript.

## Ethics Statement

The study was approved by Ethics Committee of Chinese PLA General Hospital (2020263). This research was performed in accordance with the Declaration of Helsinki, China’s regulations, and Guidelines for Good Clinical Practice. Informed consent was obtained from all subjects.

## Consent

Please see Ethics Statement.

## Conflicts of Interest

The authors declare no conflicts of interest.

## Data Availability

The data that support the findings of this study are available from the corresponding author upon reasonable request.
